# Marine Carotenoids and Oxidative Stress

**DOI:** 10.3390/md10010116

**Published:** 2012-01-16

**Authors:** Graziano Riccioni

**Affiliations:** 1 Cardiology Unit, San Camillo de Lellis Hospital, via Isonzo, Manfredonia, Foggia 71043, Italy; Email: griccioni@hotmail.com; Tel.: +39-0882-227022; Fax: +39-0882-227022; 2 Human Nutrition, Department of Biomedical Science, via Dei vestini, University G, D’Annunzio, Chieti 66013, Italy

**Keywords:** marine carotenoids, oxidative stress, cardiovascular disease, prevention, reactive oxygen species, coronary artery disease

## Abstract

Oxidative stress induced by reactive oxygen species plays an important role in the etiology of many diseases. Dietary phytochemical products, such as bioactive food components and marine carotenoids (asthaxantin, lutein, β-carotene, fucoxanthin), have shown an antioxidant effect in reducing oxidative markers stress. Scientific evidence supports the beneficial role of phytochemicals in the prevention of some chronic diseases. Many carotenoids with high antioxidant properties have shown a reduction in disease risk both in epidemiological studies and supplementation human trials. However, controlled clinical trials and dietary intervention studies using well-defined subjects population have not provided clear evidence of these substances in the prevention of diseases. The most important aspects of this special issue will cover the synthesis, biological activities, and clinical applications of marine carotenoids, with particular attention to recent evidence regarding anti-oxidant and anti-inflammatory properties in the prevention of cardiovascular disease.

## Introduction

Oxidative stress (OS) and chronic inflammation are the major pathophysiological factors contributing to the development of cardiovascular diseases (CVD), such as hypertension, diabetes and atherosclerosis. Accumulating evidence suggests that a compromised antioxidant system can lead to excessive OS in cardiovascular related organs, resulting in cell damage and death [1].

Emerging evidence suggests that interventions, including nutrition, pharmacology, and physical exercise, may activate expression of cellular anti-oxidant systems and play a role in preventing inflammatory processes in CVD [2]. For these reasons new effective interventions, based on nutrition, aimed at targeting OS and chronic inflammation, may induce an important protection from CVD [3,4].

Advances in pathophysiological research suggest that CVD represent a *continuum* of *pathophysiological processes* that advance from local redox imbalance to endothelial dysfunction, endothelial inflammation, and excessive vascular remodeling. Consequent cell damage contributes to atherosclerosis, coronary artery disease (CAD), stroke and myocardial infarction [5,6]. In particular CVD are associated with increased production of reactive oxygen species (ROS) and compromised endogenous anti-oxidant defense systems (superoxide dismutases (SODs), heme oxygenase-1 (HO-1), NAD(P)H quinone oxidoreductase-1 (NQO-1), catalase, and thioredoxin). OS is tightly regulated by a balance between production and removal of ROS. A compromised anti-oxidant defense system can lead to excessive oxidative stress and ultimately result in cell damage [7]. 

The nutritional prevention of atherosclerosis with the use of natural antioxidants represents an important new frontier in the prevention and treatment of CVD. There is evidence that the production of oxidized LDL (LDL_OX_) can be counteracted by the activity of dietary free radical electron (“antioxidant”) acceptor molecules (such as β-carotene and ascorbic acid), which sequester free radical electrons and prevent the oxidation of LDL particles [8]. Many foods typical of the Mediterranean Diet (such as olive oil, red wine, fruits, and vegetables) contain a mixture of phytonutrients that are both water-soluble and lipid-soluble and can enhance antioxidant capacity throughout the organism, and variations of that dietary regimen are even more effective. For example, several naturally-occurring phytochemicals with antioxidant actions have been associated with the prevention of atherosclerosis, including the carotenoids, lycopene, lutein and astaxanthin, and glabridin, the major isoflavan obtained from licorice roots, even if this evidence is derived from studies presenting an important limitation due to the small number of subjects [9].

Although a large body of research has focused on individual or small numbers of antioxidants, increasing circulating antioxidant capacity through increased consumption of antioxidant-rich fruits and vegetables is protective against cardiovascular disease. The available scientific evidence indicates that the link between oxidative stress, a proinflammatory systemic environment and cardiovascular disease is strong [10]. This conclusion can provide increased motivation for dietary improvements that shift the risk equation away from premature death and toward increased longevity and enhanced quality of life.
